# Investigation of Molten Metal Infiltration into Micropore Carbon Refractory Materials Using X-ray Computed Tomography

**DOI:** 10.3390/ma14123148

**Published:** 2021-06-08

**Authors:** Jakub Stec, Jacek Tarasiuk, Sebastian Wroński, Piotr Kubica, Janusz Tomala, Robert Filipek

**Affiliations:** 1 Faculty of Materials Science and Ceramics, AGH University of Science and Technology, Al. Mickiewicza 30, 30-059 Kraków, Poland; stec@agh.edu.pl; 2 Faculty of Physics and Applied Computer Science, AGH University of Science and Technology, Al. Mickiewicza 30, 30-059 Kraków, Poland; tarasiuk@agh.edu.pl (J.T.); wronski@fis.agh.edu.pl (S.W.); 3 Tokai COBEX Polska sp. z o. o., ul. Piastowska 29, 47-400 Racibórz, Poland; piotr.kubica@tokaicobex.com (P.K.); janusz.tomala@tokaicobex.com (J.T.)

**Keywords:** blast furnace, carbon refractories, molten metal infiltration, X-ray computed tomography

## Abstract

The lifetime of a blast furnace (BF), and, consequently, the price of steel, strongly depends on the degradation of micropore carbon refractory materials used as lining materials in the BF hearth. One of the major degradation mechanisms in the BF hearth is related to the infiltration and dissolution of refractory materials in molten metal. To design new and more resilient materials, we need to know more about degradation mechanisms, which can be achieved using laboratory tests. In this work, we present a new investigation method of refractory materials infiltration resistance. The designed method combines a standard degradation test (hot metal penetration test) with X-ray computed tomography (XCT) measurements. Application of XCT measurements before and after molten metal infiltration allows observing changes in the micropore carbon refractory material’s microstructure and identifying the elements of the open pore structure that are crucial in molten metal infiltration.

## 1. Introduction

While blast furnace (BF) technology remains the main method of obtaining crude iron (known also as pig iron), the cost of steel strongly depends on the lifetime of the BF [1]. Its working time is mainly limited by the degradation of refractory materials, especially when used in the BF hearth area, where liquid metal gathers. It is a consequence of the fact that damage to BF hearth walls cannot be repaired without stopping the work of the whole furnace [2,3]. In that crucial zone, carbon and graphite refractory materials are mainly used, due to their unique properties, such as thermal stability, high thermal conductivity, corrosion resistance, and non-wettability by molten metal and slag [4].

The complexity of the processes occurring inside the BF results in various degradation mechanisms present in each zone of the BF. In general, they can be divided into three groups: mechanical wear (e.g., abrasive wear of descending solid burden, erosion resulting from ascending dust-laden gases and from the molten metal flow), thermal wear (e.g., thermal shocks caused by the tapping cycles and thermomechanical stresses resulting from temperature gradients), and chemical wear, which is a result of the interactions at high temperatures between various compounds present in the BF and lining materials [5]. In the BF hearth, carbon and graphite refractory materials are mainly subjected to chemical wear, which includes various degradation mechanisms, such as alkali [6] and zinc attack [7], carbon monoxide deterioration [8], erosion, dissolution, and infiltration of refractories by molten crude iron [9,10]. All of them do not occur separately as independent processes, but they contribute to the overall degradation of the BF hearth, which changes the temperature distribution and further accelerates the degradation process [11,12]. 

To design new, more resilient refractory materials, we need to know more about degradation mechanisms. One of the possible sources of such information is postmortem sample analysis. However, this method is limited by sample availability and provides information about the sum of all degradation mechanisms [13]. An alternative approach, which can be used to evaluate new materials and their resistance to various degradation mechanisms are laboratory tests. They are more available than postmortem samples, less expensive, and much quicker, which makes them an essential tool in the process of designing and evaluating new refractory materials. Degradation in contact with molten metal, which is a major degradation mechanism of carbon and graphite materials, can be investigated using the most common tests, e.g., sessile drop, immersion, and crucible, or more complex ones, such as rotatory tests or with induction furnaces [14]. All of these focus on chemical degradation (i.e., dissolution of refractory materials) and/or mechanical erosion, thus on the processes which occur on the surface of refractory materials. However, it is commonly accepted that in the actual BF hearth, molten metal might penetrate refractory bricks up to the 1150 °C isotherm [15]. To investigate the materials resistance to molten metal infiltration, a new test—the hot metal penetration (HMP) test—was designed. It is a modification of the crucible test, in which the carbon-saturated crude iron is forced to infiltrate the tested material by the elevated pressure created inside the crucible by the flow of argon [16]. In the standard HMP procedure, after the infiltration, the crucible is investigated to evaluate if there was a leakage of molten metal (the material can pass or fail the HMP test). To analyze the infiltration zone and metal flow paths, the crucible is cut into smaller pieces and observed via optical or scanning electron microscopy [17]. While this approach provides qualitative information about material infiltration resistance, we are not able to identify the initial elements of material microstructure which are essential in the process of molten metal infiltration. 

To better understand the dependence between material microstructure and their degradation resistance, we need to compare the 3D microstructures, before and after the degradation process. This can be achieved by using nondestructive investigation methods, among which X-ray computed tomography (XCT) is a promising candidate. XCT can be used to investigate the microstructure of multiphase materials [18,19,20] and pore structure [21,22]. Based on the XCT measurements, methods for determining parameters describing pore structure—tortuosity [23] and constrictivity [24]—were developed. Only for carbon and graphite refractory materials, XCT has been used to investigate the pore structure of micropore carbon materials [25], microstructure and tortuosity analysis of carbon anodes for aluminum smelting [26,27] and for porosity investigations of carbon blocks and ramming paste used for silicomanganese production [28]. 

XCT is a powerful tool, which can be used to investigate the microstructure of composite materials before and after the chemical vapor deposition process [29] or to qualitatively describe the corrosion of steel-fiber reinforced polymer bars [30]. Moreover, XCT can be used in the in situ observation of degradation processes, such as SiC_f_/SiC composite fatigue testing [31] or steel bar corrosion in cementitious matrix [32].

In this work, we present a novel investigation method of molten metal infiltration, which combines the hot metal penetration (HMP) test with X-ray computed tomography (XCT) measurements. The new method, the XCT–HMP test, provides a unique opportunity to compare exactly the same volume of refractory material before and after molten metal infiltration. It enables the identification of the preferred infiltration paths, which could be used to correlate the material microstructure with its infiltration and consequently degradation resistance.

## 2. Materials and Methods

### 2.1. Materials

The micropore carbon trial material, investigated in our work, was produced on an industrial scale by Tokai COBEX Polska sp. z o.o (Racibórz, Poland). The main raw materials were: artificial amorphous carbon grains containing silicon carbide, synthetic graphite grains, synthetic semigraphite flour, silicon and alumina powder, and coal tar pitch as a binder. The ratio between solid particles and binder was 3.64:1. The prepared raw materials composition was subjected to a two-step mixing process using the Eirich homogenizer [33]. First, the dry compounds were mixed at temperatures below 120 °C for 5 min. After that, the binder was added and the mixing process has been continued for 15 min at 150 °C. The created green paste was further molded into blocks with dimensions of 2500 × 700 × 500 mm^3^ using a vibration molding press. Formed blocks were baked in a standard ring furnace under reducing atmosphere. The maximum baking temperature was 1300 °C. From the bottom part of the carbon block, known as the foot, the crucible and sample were cut.

### 2.2. Hot Metal Penetration Test and Its Modification

#### 2.2.1. Standard HMP Test

**Figure 1:**
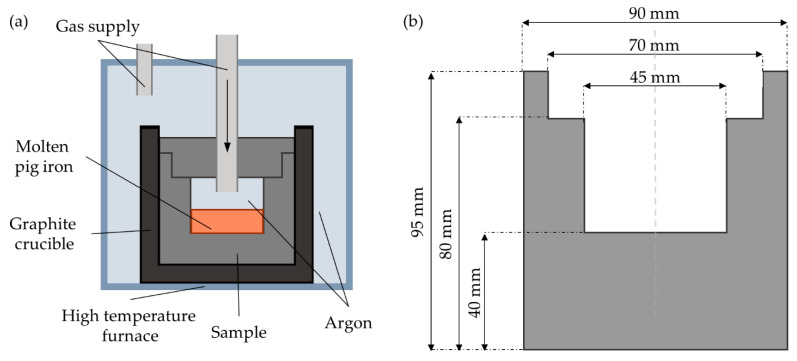
Hot metal penetration test: (**a**) scheme of HMP test and (**b**) dimensions of the HMP crucible.

The hot metal penetration test was performed using equipment designed and built in collaboration with Tokai COBEX sp. z o.o. (Racibórz, Poland), and Czylok (Jastrzębie-Zdrój, Poland). The scheme of the HMP apparatus is presented in Figure 1a. It consists of a vertical chamber furnace, accessible from the top, lined with alumina and heated by the MoSi_2_ heating element. The investigated material is machined in the form of a crucible, of which dimensions are presented in Figure 1b. Into the crucible, 150 g of carbon saturated crude iron are placed in the form of granules. The typical composition of crude iron used in the HMP tests is presented in Table 1. 

**Figure 2:**
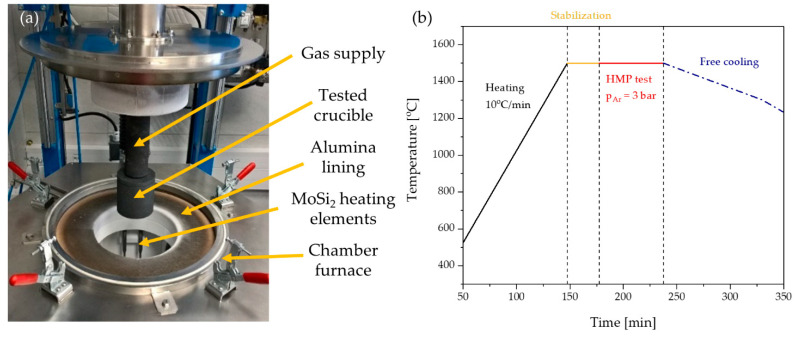
(**a**) Photo of HMP apparatus. (**b**) Heating curve during the HMP test.

The tested crucible is tightly closed with a lid made of the same material. The tight connection is ensured by a gasket made of expanded graphite foil. The lid has a hole in the center—a conduit for a pipe that is used to deliver argon inside the crucible. An external graphite crucible is used to protect the furnace heating elements in case of molten metal leakage from the tested crucible. The prepared crucible is placed in the furnace chamber by screwing in a vertical graphite tube, which serves as a gas supply (Figure 2a). The weight of the crucible is continuously monitored, therefore possible metal leaks can be detected during the ongoing process. 

The whole system is annealed from room temperature to 1500 °C at a heating rate of 10 °C/min. After reaching the desired temperature, the whole system is maintained for 30 min to stabilize it. Then, an elevated pressure of 3 bars is created inside the crucible using argon flow to force the molten metal infiltration. The crucible was kept at an elevated temperature and pressure for 60 min. After that, the whole system is freely cooled. The heating curve during the standard HMP test is presented in Figure 2b. During all stages of the HMP process, the ambient atmosphere in the furnace is maintained by the flow of argon (10 L/min) to avoid the oxidation of the carbon crucible and gas supply. 

#### 2.2.2. Modification—XCT–HMP Test

**Figure 3:**
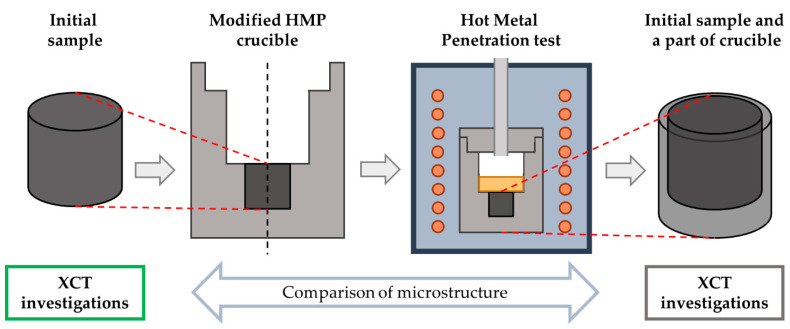
Scheme of the designed new investigation method—the XCT–HMP test.

The modified HMP test (XCT–HMP) was designed based on the idea of observing the 3D microstructure of exactly the same volume before and after molten metal infiltration. One of the major aspects of XCT measurement is its resolution, which strongly depends on the size of the sample. According to Silva et al. [6], molten crude iron can penetrate pores with diameters of up to 5 µm in actual BF hearth. Our previous investigations [25] showed that, for cylindrical samples (Ø = 25 mm, h = 25 mm), we are able to achieve a voxel size of 16 × 16 × 16 µm^3^ and observe continuous pore structures through the whole height of the sample. Consequently, the investigation of the whole HMP crucible will result in even lower resolution. To bypass this limitation, we decided to focus on a smaller sample. The scheme of the XCT–HMP test is presented in Figure 3. From the same block of micropore carbon material, a cylindrical sample (Ø = 25 mm, h = 25 mm) and a modified HMP crucible were cut. Compared to the standard HMP crucible, the new one had a hole in the center of its bottom of which the dimensions matched the size of the cylindrical sample. The sample was investigated using XCT, as described in the next section. After the measurement, the sample was inserted into the crucible. The gap between the sample and crucible was filled with sodium silicate sealing paste Firecement HT 1500 °C sealant (SOUDAL, Czosnów, Poland), which ensured a tight connection between them. Then, the modified crucible was used in the HMP procedure described in the previous section. After the test, the cylinder (Ø = 30 mm, h = 40 mm) was cut from the center of the crucible bottom. This new sample, which included the initial sample and part of the crucible’s bottom, was investigated via XCT using the same parameters. 

### 2.3. X-ray Computed Tomography

#### 2.3.1. X-ray Computed Tomography (XCT) Measurements

The XCT measurements were performed using a Nanotom 180S (GE Sensing & Inspection Technologies GmbH, Wunstorf, Germany). The machine is equipped with a nanofocus X-ray tube with maximum 180 kV voltage. The tomograms were registered on Hamamatsu 2300 × 2300-pixel detector. During the measurements, a tungsten target was used. The polychromatic beam was filtered using a 0.5-mm copper filter. The working parameters of the X-ray tube were I = 250 μA and V = 70 kV. A total of 1600 projections were taken with 4 integrations for each exposition. The total time of measurement was around 120 min. The reconstructions of the measured objects were done with the aid of the proprietary GE software, datosX ver. 2.1.0, using the Feldkamp algorithm for cone beam X-ray CT [34]. The final resolution of the reconstructed object was 16 μm. The postreconstruction data treatment was performed using VGStudio Max 2.1 software (Volume Graphic GmbH, Heidelberg, Germany) [35].

#### 2.3.2. Processing of XCT Data

The XCT results in the form of 16-bit stacks of 2D cross-section images were imported into open source ImageJ software [36]. In the first step, brightness and contrast were adjusted. Then the porosity of the initial sample and metal inclusions were separated using appropriate thresholds. Next, the continuous pore and metal structures were separated using the Flood Fill 3D tool in ImageJ. Volume fractions of both structures were calculated using Voxel Counter tool for each slice separately and then averaged for the whole volume. Local thicknesses of pore and metal structures were calculated using the thickness option in ImageJ plugin—BoneJ [37]. The 3D visualizations of samples, pore, and metal structures were created using Simpleware ScanIP software (Synopsys, San Jose, CA, USA) [38]. Details of each XCT data processing step were described in [39].

### 2.4. Microstructure and Chemical Composition Analysis

After HMP test and XCT measurements, the sample (Ø = 30 mm) was cut into 5 mm discs, ground, and polished for microstructure and chemical composition analysis. They were carried out using a scanning electron microscope NOVA NANO SEM 200 (FEI, Acht, The Netherlands) equipped with an energy dispersive spectrometer (EDAX, Tilburg, The Netherlands). Samples were observed in backscattered electron (BSE) mode. The applied energy of the electron beam was 18 keV.

## 3. Results

**Figure 4:**
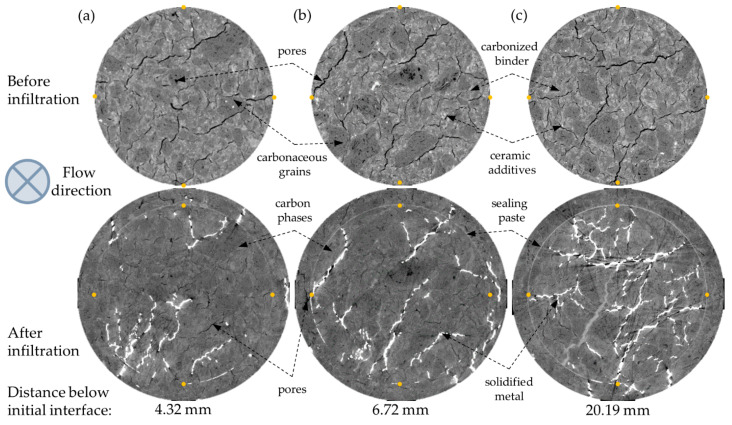
Two-dimensional cross-sections in the plane perpendicular to the molten metal flow of the sample before (top row) and after the HMP test (bottom row) at various distances from the initial molten metal/sample interface: (**a**) 4.32 mm (**b**) 6.72 mm and (**c**) 20.19 mm.

**Figure 5:**
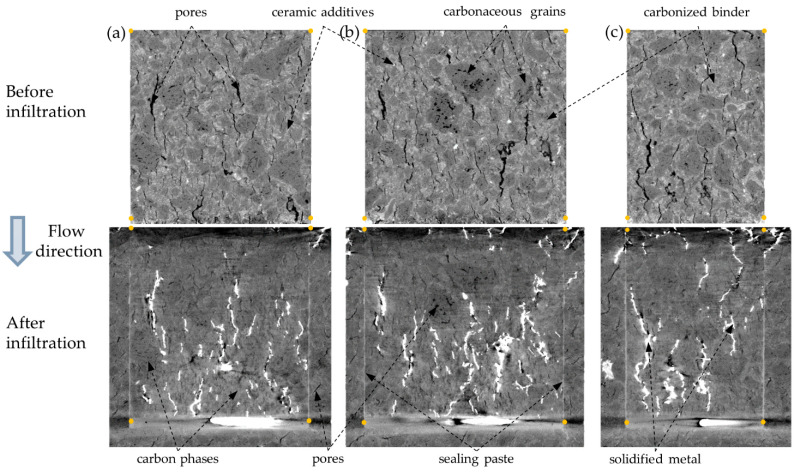
Various 2D cross-sections (**a**–**c**) in the plane parallel to the molten metal flow of the sample before (top row) and after the HMP test (bottom row).

The results of XCT investigations are presented in the form of two types of 2D cross-sections: perpendicular (Figure 4) and parallel (Figure 5) to the molten metal flow direction. The cross-sections of the sample before metal infiltration are presented in the top row, while the cross-sections after the infiltration are presented in the bottom row. In the sample before the HMP test (top row), four phases can be distinguished: pores (black), carbonaceous grains (dark grey), carbonized binder (light grey), and ceramic additives (white). In the sample after the HMP test (bottom row), due to the bigger X-ray beam, the attenuation of heavy solidified metal and consequently its very high brightness [40], the contrast between carbon and ceramics phases in the micropore carbon material is much lower than in the initial sample. As a result, it makes it difficult to distinguish between carbonaceous grains and carbonized binder, as well as between ceramic additives and solidified metal. Four phases can be easily distinguished in the sample after the HMP test: pores (black), carbon phases in the micropore carbon material (dark grey), solidified metal (white), and the sealing paste (light grey) in the gaps between the initial sample and the surrounding crucible. Comparing the sample microstructure before and after HMP test, it can be seen that the major part of the initial porosity was filled with the molten metal. Pores in the lower part of the sample were impregnated with metal to a greater extent than in the upper part of the sample. Portions of molten metal infiltrated below the initial sample in the zone, which presumably was not filled with the sealing paste (Figure 5). The sealing paste was used as a junction material, which provided a tight connection between the crucible and sample in the major part, which prevented the infiltration through the gap between sample and the crucible instead of the sample volume. However, the zones which were not filled with the sealing paste can be observed, especially in the upper parts of the sample (Figure 4b,c)—during the HMP test they were filled with molten metal. Moreover, not only the gap between the sample and crucible was filled with sealing paste, but also a part of the open pore structure in the micropore carbon material (Figure 4c). 

Analyzing the 2D cross-sections after the HMP test, the artifacts can be seen both in the upper (Figure 5, dark areas in the top row) and lower part of the sample (Figure 4c), in the zones where there was a higher amount of molten metal. As a result, the phase segmentation of phases other than bright metal in those areas was inaccurate.

**Figure 6:**
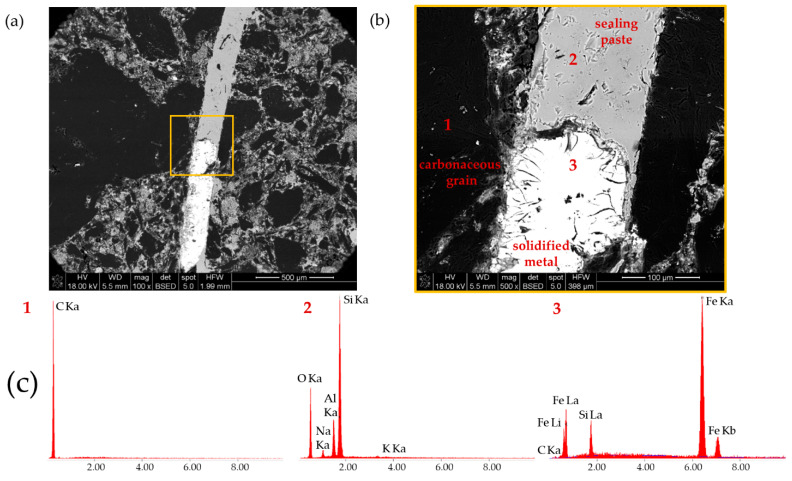
(**a**) SEM image of crucible sample and junction between them after metal infiltration; (**b**) SEM image of junction and solidified metal with marked points for EDS analysis; and (**c**) results of EDS point analysis.

To verify if the brighter grey phase in XCT investigation after the HMP test was the actual sodium silicate sealing paste, the sample after metal infiltration was cut into the discs and then further prepared for the scanning electron microscope investigations by grinding and polishing. The microstructure of the crucible sample and the junction between them are presented in Figure 6a. In the observed area, the junction did not fill the whole gap and a part of it was impregnated by the molten metal, which was also visible in the XCT observations (Figure 4, bottom row). The chemical composition analysis of phases in the gap between the sample and crucible were performed in the area marked with an orange rectangle (Figure 6b). Results of the EDS analysis are presented in Figure 6c. In the solidified metal (point 3), we can observe the characteristic peaks for Fe, Si, and C—the three main elements of crude iron used in HMP test (Table 1). The point analysis of the bright grey phase (point 2), shows that it is mainly composed of Si, Al, and O, with small amounts of Na and K, which proves that the bright grey phase observed in the XCT data is the sodium silicate sealing paste.

**Figure 7:**
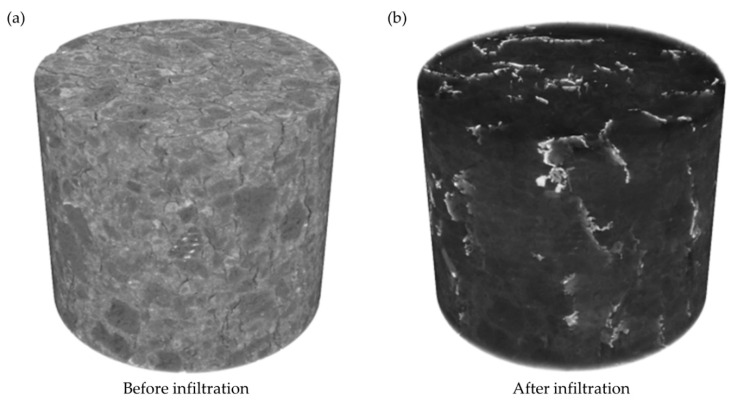
Three-dimensional visualization of samples: (**a**) before and (**b**) after the HMP test.

To evaluate the influence of molten metal on the microstructure of micropore carbon materials, for both XCT measurements, the same volumes, representing the sample before and after molten metal infiltration were cut. The diameters of the analyzed volumes were equal to the diameter of the initial sample (Ø = 25 mm), while their heights were lower, h = 20.8 mm. The chosen samples’ height was lower than that of the initial sample (h = 25 mm), due to the fact that part of the crucible bottom was dissolved during the HMP test and the artifacts in the upper part of the sample made the proper phase segmentation of areas close to the initial molten metal/carbon material interface impossible. The 3D visualizations of the chosen volumes for the analysis are presented in Figure 7. 

**Figure 8:**
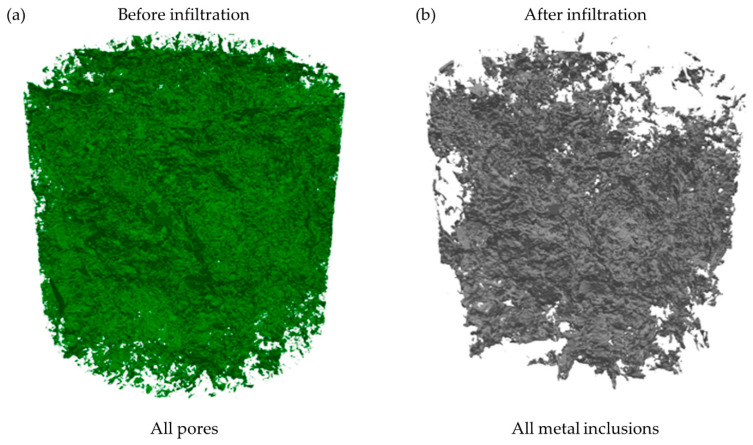

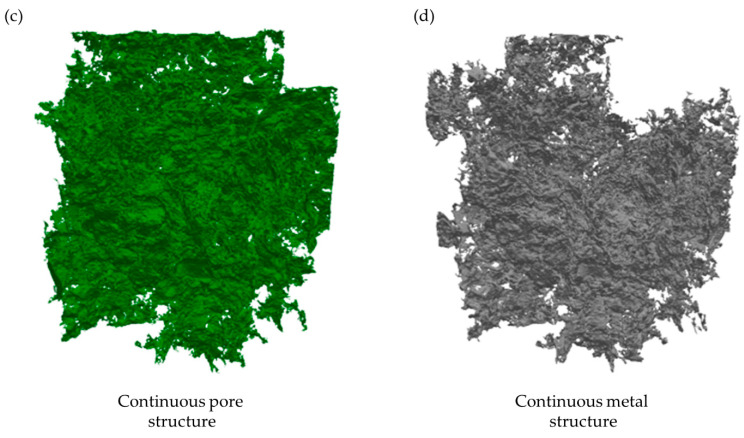
Visualization of (**a**) all pores in the initial sample, (**b**) all metallic inclusions in the sample after the test, (**c**) continuous pore structure, and (**d**) continuous metal structure.

**Figure 9:**
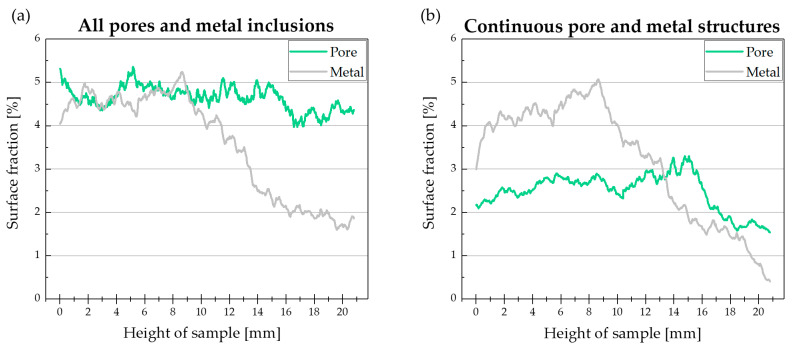
Surface fractions of pores and metal inclusions as a function of the sample height: (**a**) all pores and all metal inclusions and (**b**) continuous pore and metal structure.

In the next step, the pores in the initial sample and metal inclusions were separated by applying suitable thresholds to the analyzed images. The separated pores and metal inclusions are presented in the form of 3D visualization in Figure 8a,b. The volume of all pores was 474 mm^3^, corresponding to a volume fraction (porosity) of 4.64% (Table 2). The volume of all metal inclusions measured by XCT equaled 366 mm^2^, which means that the volume fraction of metal was smaller—3.59% (Table 2). The volume of all metal inclusions was approx. 23% smaller than the volume of all pores. The volumes of pores and metal inclusions were measured for each slice separately, which enabled the presentation of surface fractions as a function of sample height (Figure 9a). The distribution of pores in the initial sample was throughout the whole height of the sample, which resulted in low standard deviation (σ, Table 2). In the case of metal inclusion, the distribution was much more nonuniform. The fraction of metal in the lower part of the sample (between 0 and 10 mm) was more than 4%, while in the upper part was constantly decreasing to 2% at the top sample boundary. This resulted in a much higher standard deviation, approx. 4.5 times higher than for all pores.

Despite the fact that XCT allows measuring the sum of open and closed pores, only open pores contribute to molten metal transport. Thus, in the next stage, the continuous pore and metal structures were separated using the Flood Fill 3D tool. The continuous structures are presented in Figure 8c,d. 3D visualization of continuous pore and metal structures in form of GIF files are presented in Supplementary Materials. The volume of the continuous pore structure was approx. 2 times lower than the volume of all pores, which resulted in a 2.48% volume fraction. Its distribution was less uniform than the distribution of all pores (Figure 9b), which resulted in the higher standard deviation (0.42%). In the case of the continuous metal structure, its volume was also smaller than the volume of all metal inclusions, but the difference was much smaller (approx. 12%). The decrease in metal structure volume was a consequence of the small inclusions close to the top and bottom sample’s boundaries which were disconnected from the main metal structure (Figure 8b,d and Figure 9). It resulted in a slightly higher standard deviation (1.33%) than for all metal inclusions (1.19%) The volume fraction of the continuous metal structure was 3.19%, which was 29% larger than the continuous pore structure (Table 2).

The morphology of the pores and metal inclusions was described by the measurements of local thicknesses, presented in Table 3. The average pore thickness of all pores was 115 µm, while for all metal inclusions it was 65% higher (190 µm). The thickness distribution was more uniform for pores (σ = 54 µm) than for the metal inclusion (σ = 84 µm). Comparing the maximum local thicknesses, it can be seen that it was 20% higher for all metal inclusions (843 µm) than for all pores (703 µm). The maximum thicknesses were approx. 6 times higher for pores and 4.4 times higher for metal inclusions than the average thickness. 

In the continuous structures, the average thicknesses were higher, especially for the pore structure (135 µm), which resulted in a lower difference between pores and metal—44%. The thickness of the pore structure was slightly more even. The biggest differences were observed in the maximum local thicknesses. While for the continuous metal structure it was the same value like for all metal inclusions, the maximum thickness of the continuous pore structure was much smaller than for all pores (410 µm), and the maximum thickness for continuous metal structure was 2 times higher than for the continuous pore structure.

## 4. Discussion and Conclusions

Our research showed that XCT can be successfully applied to observe the same volume of refractory material before and after molten metal infiltration. Despite the fact that, according to Silva et al. [6], in the analyzed system (which simulates the conditions inside the actual BF hearth) pores up to 5 µm, which can be infiltrated by molten metal, our investigation with voxel size 16 × 16 × 16 µm^3^ allowed us to observe the continuous metal structures. The infiltration paths created during the HMP test were clearly visible in the XCT measurements. The small metal inclusions, disconnected from the main metal structure were observed, which resulted in the difference between the volume fraction of all metal inclusions (3.59%) and the continuous metal structure (3.19%). This difference can be an effect of the applied resolution (larger than the minimum diameter of accessible pores), which as a consequence might result in a lack of ability to observe small connections between the continuous metal structure and separated metal inclusions. However, it is also possible that those crude iron particles were separated from the main metal body. To answer, if they were connected or not, additional XCT measurements with better resolutions are required. 

Total and continuous porosities measured by XCT in the initial sample, are much lower than the open porosities measured for this particular micropore carbon material (produced by Tokai COBEX sp. z.o.o., Racibórz, Poland) using other methods, i.e., mercury intrusion porosimetry (16.62 ± 1.09%) or helium porosimetry (18.66 ± 0.70%) [41]. Comparing with the XCT measurements of other carbon refractory materials, the measured total porosity was higher than for the carbon anodes (1.96–3.46%) investigated by Rørvik et al. [27], while the continuous porosity was much lower than for amorphous carbon blocks (6–7%) investigated by the Steenkamp et al. [28]. However, it should be noted that those measurements were performed for XCT investigations with different voxel sizes (7.3 × 7.3 × 7.3 µm^3^ [28] and 27 × 27 × 27 µm^3^ [27]) than in this study. Results showed that even using this relatively high resolution (16 × 16 × 16 µm^3^), we are able to observe the elements of continuous pore structure, which serve as a preferred flow path for molten metal infiltration. This observation is in agreement with our previous investigations [25].

The presented modification of hot metal penetration test not only allowed us to observe the penetration of pore structure in micropore carbon materials but also observe the process of carbon material degradation in molten metal. The volume of the continuous metal structure (3.19%) was larger than the volume of the continuous pore structure (2.48%), which indicates that two additional processes occurred during the test: (i) dissolution of carbon material surrounding the continuous pore structure and (ii) creation of a connection between pores separated in the initial sample. Those processes can be observed in local thickness analysis. The dissolution is visible in the increase of the average local thickness from 135 µm (pores) to 194 µm (metal). The creation of the connection is proved by the changes in the maximum local thickness. For solidified metal, the maximum thickness was 843 µm (both for all metal inclusions and continuous metal structure), while for pores there was a significant difference. In total porosity, the maximum thickness was 703 µm and in the continuous pore structure it was 410 µm. Thus, the continuous pore structure was not only filled with molten metal, but it also has become connected with the largest pore present in the investigated sample. The difference in maximum pore (703 µm) and metal (843 µm) thicknesses is a consequence of carbon material dissolution in molten metal.

The application of the sodium silicate sealing paste provided a tight and stable connection between the sample and the crucible. As a result, the major molten metal infiltration occurred through the sample volume, instead of the gap between it and the crucible. However, the entire gap was not filled with sealing paste, and in certain areas the space between the sample and the crucible was filled with molten metal. Simultaneously, part of the sample pore structure was infiltrated by the sealing paste. While it does not have a major influence on the whole HMP test, in the future, the method of applying silicone has to be optimized to ensure an even more tight connection between the sample and the crucible.

In sum, a new method for molten metal infiltration investigations was designed and successfully tested. Thanks to the application of X-ray the computed tomography method enables the investigation of the mechanism of carbon refractory materials degradation by comparison of 3D microstructure before and after infiltration to observe the changes inflicted by the molten metal flow. In the future, we are planning to optimize the infiltration time to observe different infiltration zones. The XCT–HMP test will be used to verify the evolutionary two-phase flow model in 3D geometry coupled with selective dissolution of carbon phases.

## Figures and Tables

**Table 1:** Chemical composition of crude iron used in the HMP test.

Crude Iron Composition [wt.%]							
Fe	C	Si	Mn	P	S	Ti	Zn
94.619	4.780	0.400	0.085	0.073	0.024	0.014	0.005

**Table 2:** Volume fraction, standard deviation (σ), and volume (V) of pores and metal inclusions.

All Pores and Metal Inclusions	Volume Fraction [%]	Increase	σ [%]	V [mm^3^]
Pores	4.64	−23%	0.27	474
Metal	3.59		1.19	366
**Continuous Pore and Metal Structures**	**Volume Fraction [%]**	**Increase**	**σ [%]**	**V [mm^3^]**
Pore	2.48	29%	0.42	253
Metal	3.19		1.33	326

**Table 3:** Local thicknesses of the pores and metal inclusions: average local thickness ${\bar{d}}_{th}$, standard deviation, σ and maximum local thickness $d_{th}^{max}$.

Local Thickness [µm]				
		${\bar{d}}_{th}$	σ	$d_{th}^{max}$
All	Pores	115	54	703
	Metal	190	84	843
Increase		65%		20%
Continuous structure	Pore	135	50	410
	Metal	194	85	843
Increase		44%		106%

## Data Availability

The data presented in this study are available on request from the corresponding author.

## Supplementary Materials

The following are available online at https://www.mdpi.com/article/10.3390/ma14123148/s1, Video S1: 3D visualization of continuous pore and metal structures.
