# Supplementary figures and images for: Investigation of Molten Metal Infiltration into Micropore Carbon Refractory Materials Using X-ray Computed Tomography
Source: Materials (Basel). 2021 Jun 8;14(12):3148. doi: 10.3390/ma14123148 (PMC8228681; doi:10.3390/ma14123148)

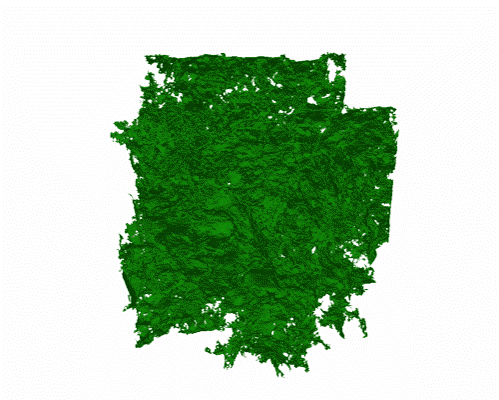

Supplement: Supplementary file 1 [file materials-14-03148-s001.zip › Continuous pore structure.gif]

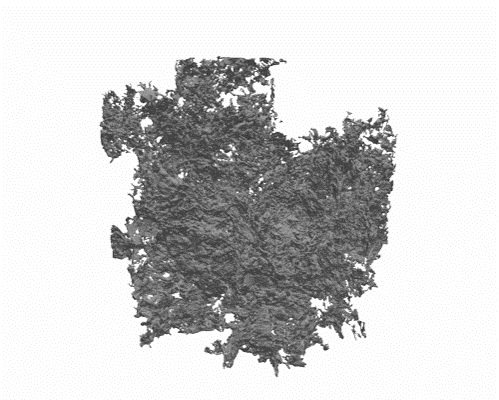

Supplement: Supplementary file 1 [file materials-14-03148-s001.zip › Continuous metal structure.gif]
